# Description of the root anatomy of the primary molars using high resolution computed microtomography (Micro-CT). An analysis of three-dimensional root canal system

**DOI:** 10.3389/fdmed.2024.1522414

**Published:** 2025-01-06

**Authors:** Benedetta Vidi, Ana María Gil-Valcarcel, Cristina Obispo-Diaz, Isabel Sanchez-Jorge, Jesús Mena-Álvarez, Juan Manuel Aragoneses-Lamas, Cristina Rico-Romano

**Affiliations:** ^1^ Faculty of Dentistry, Alfonso X El Sabio University, Villanueva de la Cañada, Spain; ^2^ Doctoral Program in Health Science, University of Alcalá, Alcalá de Henares, Spain; ^3^ Department of Surgery, Alfonso X El Sabio University, Villanueva de la Cañada, Spain; ^4^ Departments of Endodontics, Faculty of Dentistry, Alfonso X El Sabio University, Villanueva de la Cañada, Spain

**Keywords:** deciduous teeth, internal root canal, Micro-CT, primary molars, root canal morphology, Vertucci classification

## Abstract

**Background/purpose:**

This study aimed to describe the root canal morphology of primary molars using Micro-CT and analyse the three-dimensional images of the internal root canal system.

**Material and methods:**

One hundred and twenty extracted primary molars with one or more completed roots were scanned with Micro-CT. Three fixed heights of the roots were determined at axial level and the analysed criteria were: dentine thickness, diameter of roots canals, presence of lateral canals, isthmus and number of foramina. Vertucci's classification was also studied.

**Results:**

Vertucci Type I was more prevalent in palatal and mesio-buccal root of superior molar and distal root of inferior molars. Type IV was more frequent in the mesial root. Dentine thickness was studied at three levels and for all the surfaces of the root canal. In the mesiobuccal root, in the middle and apical third, the mean in the upper molars is significantly higher than in the lower molars. The mesio-lingual canal has the major mean in mesio-distal and bucco-lingual diameter at coronal third. Isthmus were found mostly in the mesial root in all three levels. The high number of foramina and lateral canals were located at apical third of the mesial and mesio-buccal roots.

**Conclusion:**

This study showed the complexity and variability of the root canal morphology of primary molars. The Micro-CT images gave important information about the internal anatomy of the primary molars.

## 1 Introduction

Temporary teeth are essential for proper human development, in addition to performing numerous and indispensable functions such as chewing, digestion and stimulation of mandibular growth (1, 2).

The main cause of pulp and periapical diseases affecting temporary and permanent teeth is caries (3), which remains a serious public health problem worldwide (4). If caries is not treated in temporary dentition, in addition to pain and premature loss of deciduous teeth, the risk of developing the disease in permanent dentition will increase (4–6). Pulpectomy is the treatment of choice for primary teeth with irreversible pulp involvement or necrotic pulp (3, 5–8). Endodontic treatment in temporary teeth aims to maintain the integrity and health of primary teeth and supporting tissues (9, 10).

Temporary teeth have different anatomical characteristics compared to permanent teeth (size, external/internal crown-root morphology), as well as duration and permanence in the mouth and in the physiological resorption processes (3, 8). We can highlight the structural differences of the dentin (8) and the external morphology of the roots, since these are longer, thinner, curved and divergent (11, 12).

The process of rhizolysis or physiological root resorption that temporary teeth undergo must be taken into account before performing pulp treatment (8). Therefore, the indications for pulpectomy are also based on the level of root resorption present and on the stage of development of the permanent tooth. Likewise, when the length of the root changes due to rhizolysis, the location of the apical foramina is also modified, which tend to be relocated more coronally (3).

The steps to follow in pulpectomy are similar to those of endodontic treatment in permanent teeth: complete removal of the altered pulp tissue, cleaning and disinfection of the pulp chamber and the walls of the root canals, and subsequent filling with a resorbable material (7, 9, 13, 14).

Maintaining root dentin thickness is essential to prevent endodontic problems such as fissures or perforations (15). The minimum dentin thickness of 1.5 mm has to be maintained along the entire root after the file preparation phase: this value is related to the root fracture resistance (13). There are not many studies in the literature evaluating changes in dentin thickness before and after endodontic treatment in deciduous teeth.

Physiological resorption of primary teeth, pathological resorption of roots, together with the continuous deposition of dentin, are the factors that drastically alter the size, shape and number of root canals, increasing the complexity of root morphology (1). Dentin causes narrowing of the canals, modifying their diameter and shape (10, 16). Depending on the type of tooth, resorption appears on the root surfaces closest to the permanent tooth: the lingual surface of the roots of single-rooted teeth and the internal surfaces of the inter-root zone of the furcation of temporary molars (17).

Currently, there is no established classification to describe root canal morphology in primary teeth (18). The Vertucci classification system is the most widely used and categorizes the root canal systems in permanent teeth (18–20). Although this classification has been used to perform studies on the morphology of the canals of primary teeth (19, 21), Ahmed et al. (22) presented a classification for the primary dentition based on their classification system for permanent teeth (20), detecting difficulties in being able to catalogue the morphological variants of the root canals and the accessories (22).

To overcome the lack of knowledge of the anatomy of temporary teeth, different techniques have been used, from dye injection to digital radiographs, cross sections, histological examinations and tissue cleaning (9). However, these are very sensitive and invasive techniques, which only present a two-dimensional image of a three-dimensional structure (9, 23). In addition, most studies focus on the number and shape of the root canal. They are few articles that are dedicated to analysing specific parameters such as the diameter of the canal, the presence and location of accessory canals and isthmus and the thickness of the dentin (24). Another limitation is the shortage and impossibility of finding intact primary teeth, without any sign of root resorption (19).

Intraoral radiography is the most commonly used technique for the diagnosis of caries and pulp lesions (2). With the advent of scanners and 3D imaging, pulp morphology studies of teeth have begun to be performed with Cone Beam Computed Tomography (CBCT) and high-resolution micro-computed tomography (Micro-CT) (16). CBCT allows the examination of the studied area in three spatial planes, eliminating the overlap with anatomical structures of no interest (19), while Micro-CT offers a reproducible, three-dimensional, and above all non-invasive technique (16). The non-destructive approach of Micro-CT allows the study of anatomy in a more precise way, since the internal anatomy can be reconstructed and observed from several angles (25), however, they cannot be carried out in *in vivo* studies (16). Its use is not very frequent to analyse the pulp anatomy of the root canals in temporary teeth (16, 18, 23, 24, 26, 27).

The aim of this descriptive study is to provide a more accurate view of the existing variants of the pulp anatomy of the root canals of primary molars through the use of Micro-CT.

## 2 Methods

### 2.1 Sample selection and preparation

One hundred and twenty primary molars were randomly collected: 20 upper first molars, 50 upper second molars, 20 lower first molars, and 30 lower second molars. Age, sex, and race were not taken into account.

Inclusion criteria were: minimum of two-thirds of root length remaining, presence of one or more roots, teeth without root canal treatment, teeth with caries or fillings. In the sample preparation phase the primary teeth were cleaned of soft tissue with a toothbrush and rinsed with running water. They were then stored separately in 0.1% thymol at room temperature (16). Photos of the sample were taken and it was decided to identify each tooth by using a letter and a number (e.g.: D1, T1, S1).

### 2.2 Scanning and reconstruction of the sample

Each tooth was dried and a maximum of 5 teeth were mounted on each fixture for scanning with a Micro-CT scanner (CT-SCAN-XT H-160, Nikon Metrology Europe, Leuven, Belgium). An isotropic resolution of 21 µm was used. The other acquisition parameters were 160 kV, 205 µA, and the scanner rotated 360°, with 354 ms per exposure, 3,015 projections with 2 frames per projection, and a 0.625-mm copper filter.

The images obtained were reconstructed with a specific software (VG MAX 2.2, Volume Graphics Gmbh, Heidelberg, Germany) that joins the axial cross sections and produces a 3D image. In the images and in the sections the internal anatomical structures of each specimen can be seen. The cross sections were saved in DICOM and STL format. For each tooth, it took one and a half to two and a half hours to scan. After scanning, the teeth were stored separately in 0.1% thymol.

### 2.3 Observation, description and study of the anatomy of root canals

Using the myVGL 2023.4 64bit viewer (Volume Graphics GmbH, Heidelberg, Germany), two observers (VB, CRR) with experience in paediatric dentistry and endodontics, performed the analysis of the 3D images and cross sections. First, three fixed heights of the roots of the primary teeth were determined at the axial level: (1) at the coronal level: 1 mm below the root furcation; (2) At the apical level, 1.5 mm from the radiographic anatomical apex. (3) At the mid-level, using the mean value between the two points described above. It was decided to use specific and fixed points to obtain precise and reproducible results for the entire sample. 1.5 mm from the anatomical apex to rule out the possibility of having minimal root resorptions.

**Figure 1:**
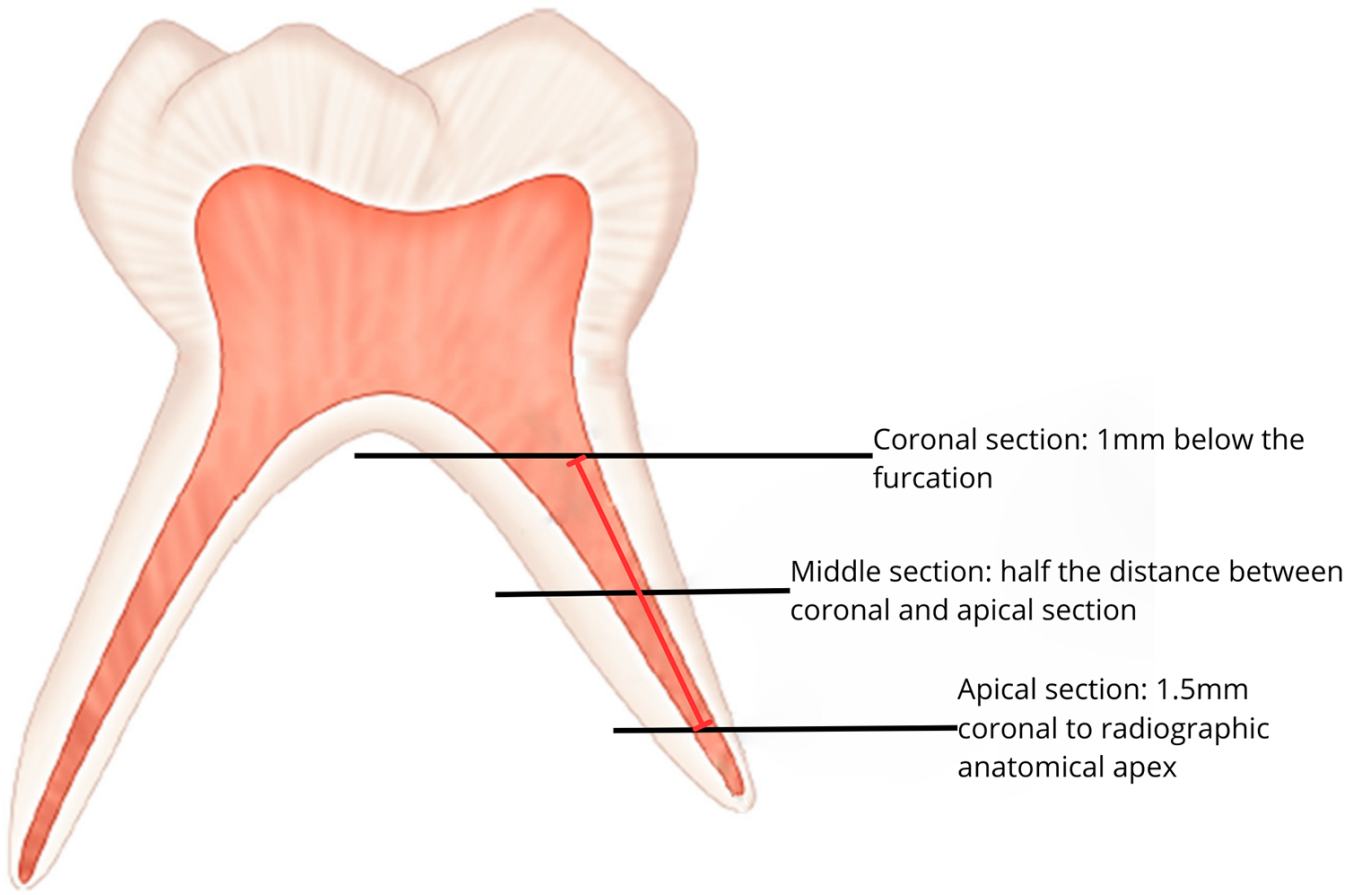
Fixed sections at which the three axial cuts have been made.

**Figure 2:**
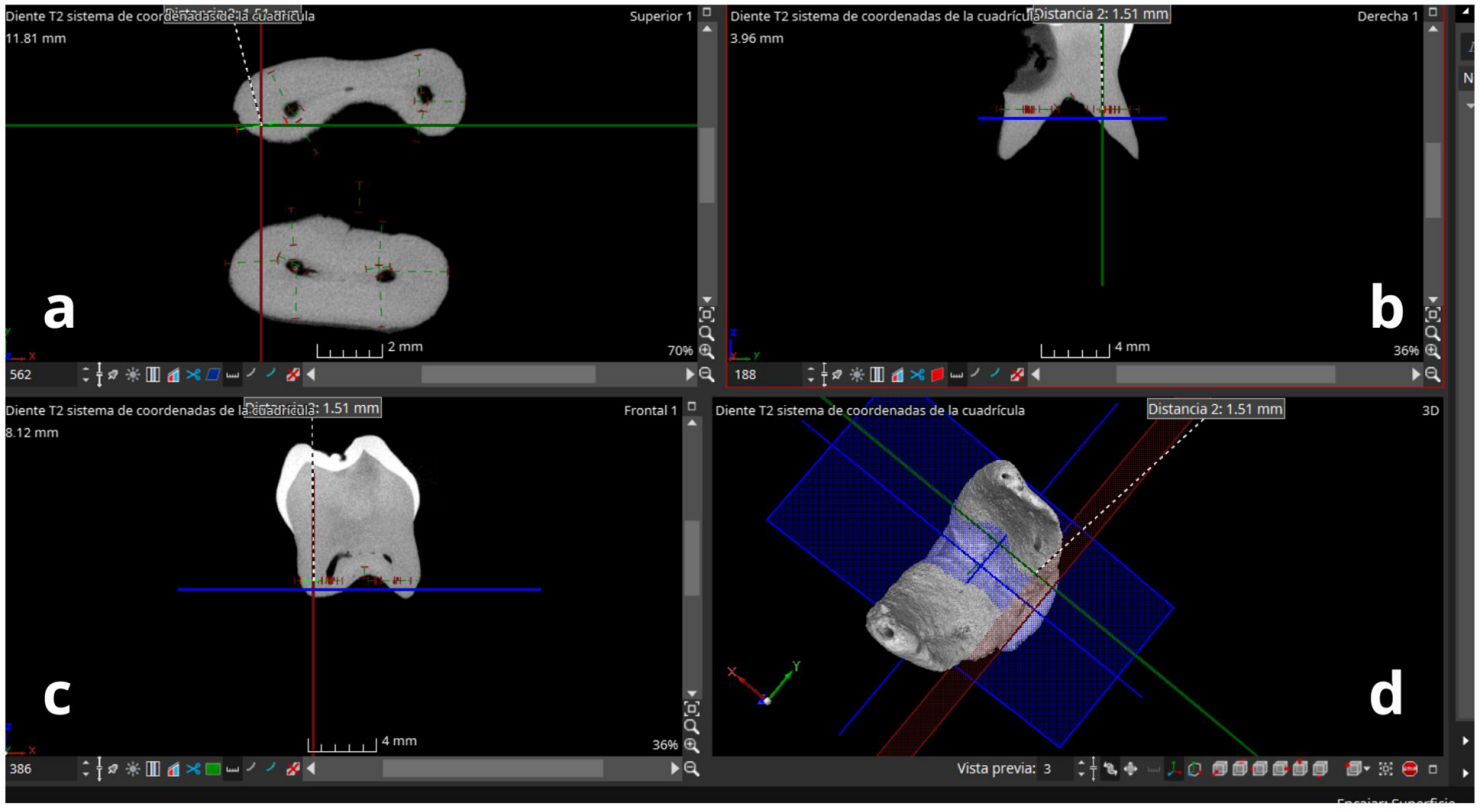
Working windows of the myVGL 2023.4 software. **(a)** Axial section; **(b)** coronal section; **(c)** sagittal section; **(d)** 3D view of the lower molar apices. Axial plane: blue; sagittal plane: green; coronal plane: red.

**Figure 3:**
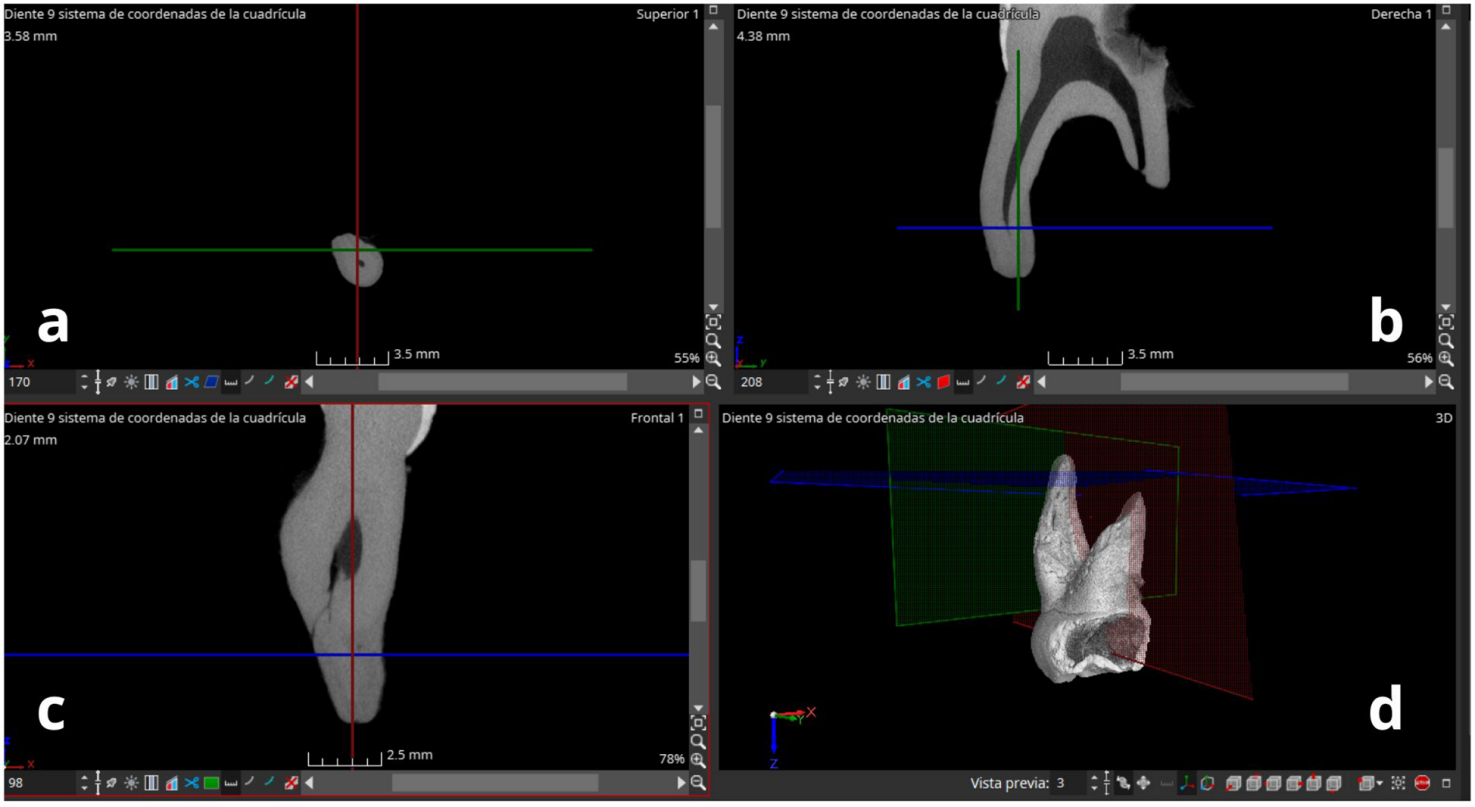
Working window of the myVGL 2023.4 software. **(a)** Axial section; **(b)** coronal section; **(c)** sagittal section with presence of lateral canals; **(d)** 3D view of the temporal molar.

**Figure 4:**
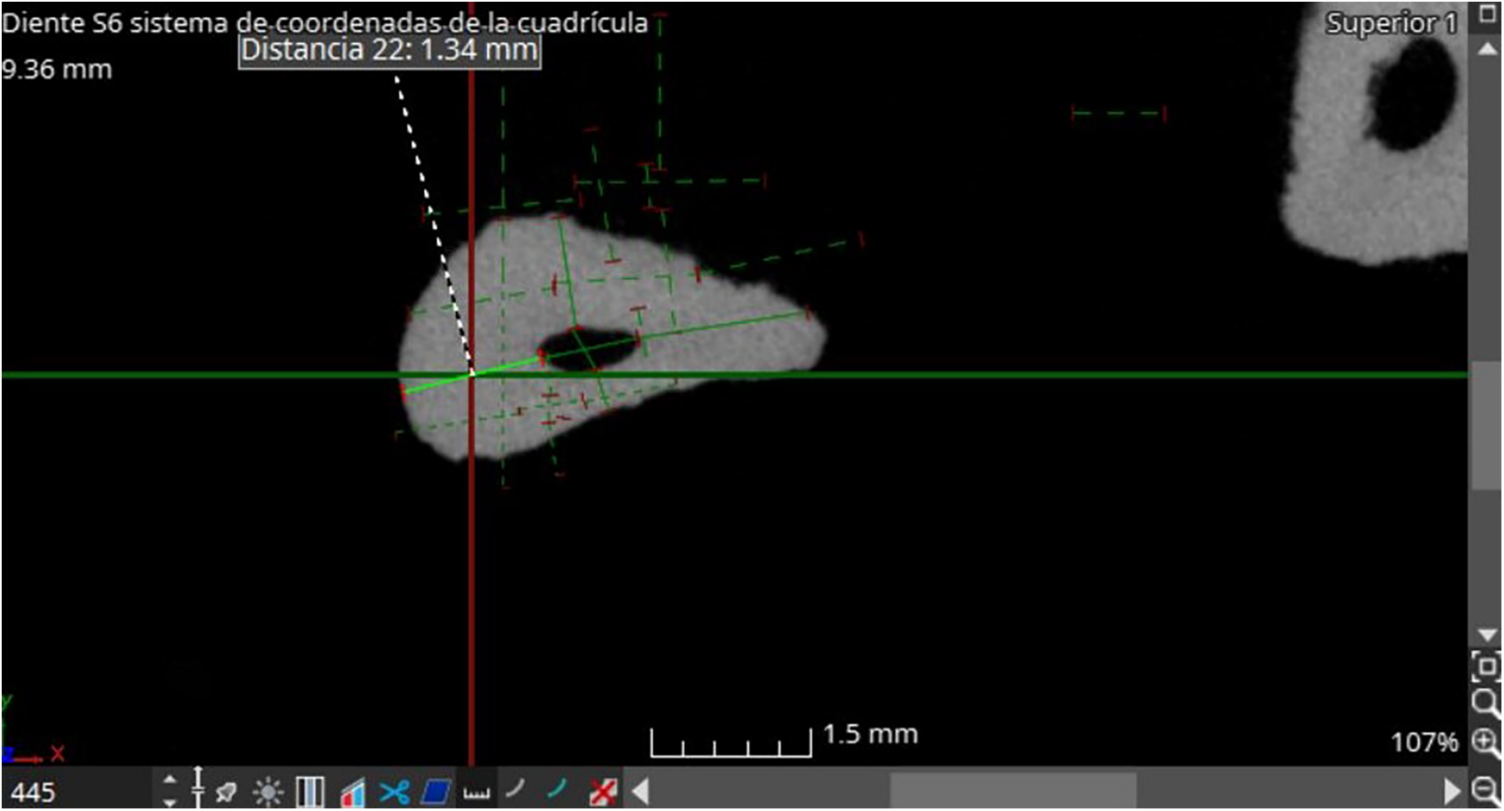
Axial section withe calculation of dentin thickness.

At these three fixed heights (Figure 1), in the axial plane, they made the following measurements:

- Root canal widths: mesiodistal (MD) and vestibulolingual (VL) (Figures 2, 3).
- Dentin thickness: vestibular, palatal/lingual, mesial and distal (Figure 4).

While, in the coronal plane, the following were evaluated:

- Canal length: the distance between the two fixed heights was calculated: apical and coronal.

**Figure 5:**
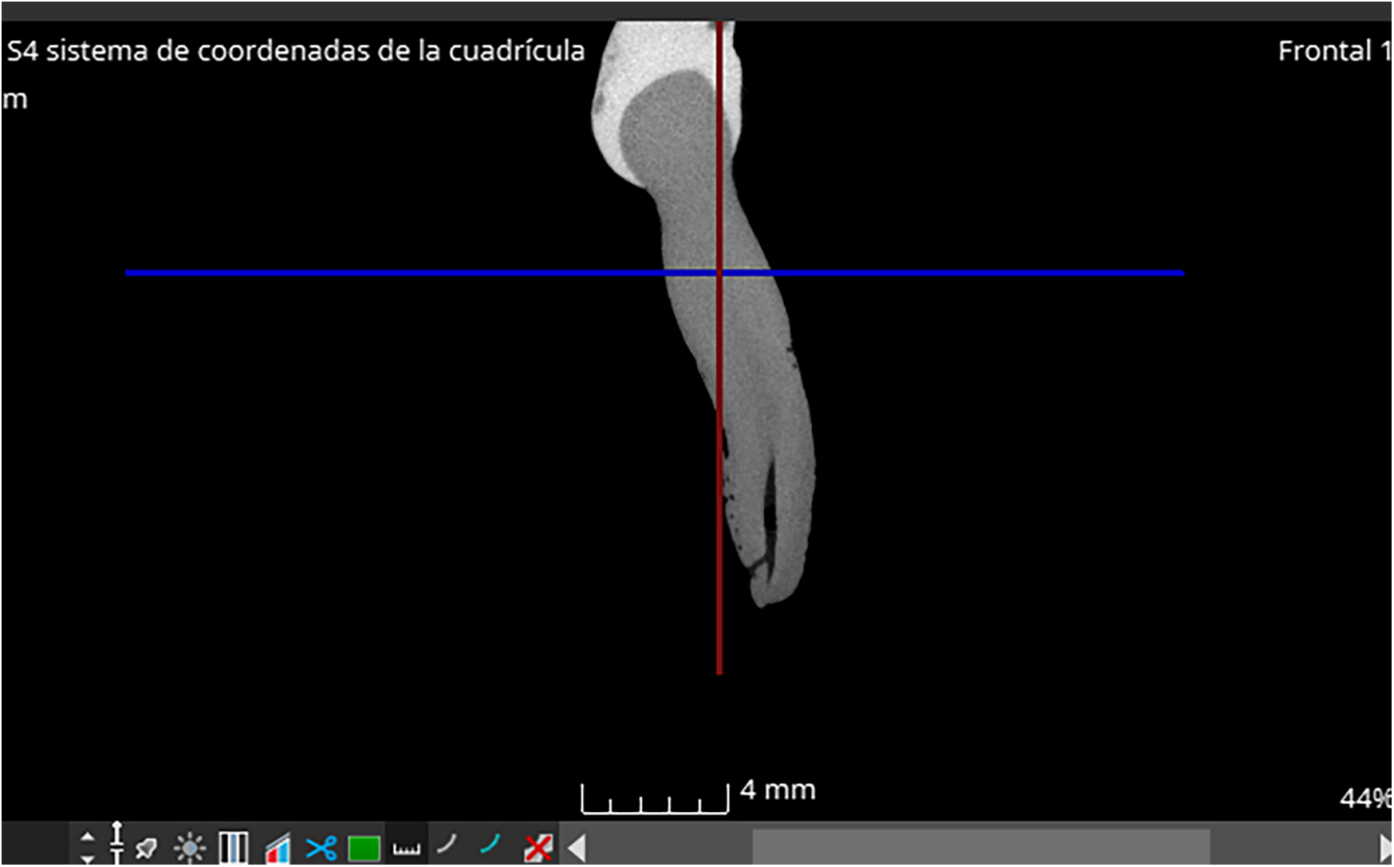
Sagittal section with an accessory canal in the apical third.

In addition, by jointly analysing the 3D images, the following parameters were collected for each tooth (Figure 5):

- Configuration and anatomy of root canals;
- Presence of lateral canals and their location;
- Presence of isthmus and their location;
- Number of foramina.

The configuration of the canal anatomy was classified according to Vertucci; canals that could not be identified in one of the parameters were classified as “unclassifiable”. The parameters chosen and studied are common to those studied by other authors (16, 24, 26–29). Likewise, the values related to dentin thickness and canal diameter are used to form a database that will be used for future studies.

### 2.4 Data analysis

The following variables were analysed qualitatively: root classification according to Vertucci, number of lateral canals, number of foramina and the appearance of isthmus, while the following variables were analysed quantitatively: the diameter of the canals, the length of the canals and the thickness of the dentin. For the descriptive analysis of the qualitative variables, frequency tables of total *n* are provided, while for the quantitative variables, means and their respective standard deviations are presented. In general, all the analyses are grouped into upper and lower molars.

Inferential analyses of comparison of means were performed in the study of dentin thickness and canal diameter. In this case, the Mann-Whitney *U* test was carried out since, due to the characteristics of the sample and the non-normality of the variables, non-parametric tests were chosen. The results will be statistically significant in values where the critical level is less than 0.05. The Kappa index was used to assess the inter-examiner reliabilit, resulting in 0.81.

All the means given in the tables represent the values in millimetres. The analyses were performed with SPSS software version 29.0.1.0.

## 3 Results

### 3.1 Dentin thickness

Table 1 analyses the dentin thicknesses of the canals based on the root and the arch to which the primary molar belongs. In the mesiobuccal root, in the middle section, the average in the upper molars (*x* = 1.21 mm) is significantly greater than in the lower molars (*x* = 0.97 mm). Likewise, it can be seen in the apical section that the average is significantly greater in the upper molars (*x* = 0.87 mm) than in the lower molars (*x* = 0.65 mm).

**Table 1:** Dentin thickness.

		Maxillary primary molars								Mandibular primary molars											
		MB ROOT				DB ROOT		P ROOT		M ROOT						D ROOT				L ROOT	
		MBC		MLC		DBC		PC		MBC		MLC		MMC		DBC		DLC		LC	
	Section	$\bar{X}(n)$	SD	$\bar{X}(n)$	SD	$\bar{X}(n)$	SD	$\bar{X}(n)$	SD	$\bar{X}(n)$	SD	$\bar{X}(n)$	SD	$\bar{X}(n)$	SD	$\bar{X}(n)$	SD	$\bar{X}(n)$	SD	$\bar{X}(n)$	SD
Buccal	Coronal	1.29 (55)	0.21			1.39 (10)	0.07	1.97 (30)	0.7	1.43 (45)	0.21					1.43 (35)	0.27			0.92 (5)	ND
	Middle	1.21* (55)	0.16			1.11 (10	0.32	0.70 (30)	0.35	0.97* (45)	0.25					1.09 (35)	0.19			0.57 (5)	ND
	Aplical	0.87* (55)	0.18			0.81 (10)	0.52	0.42 (30)	0.13	0.65* (45)	0.25					0.82 (35)	0.26			0.15 (5)	ND
Mesial	Coronal	1.12 (55)	0.96	0.90 (20)	0.15	1.32 (10)	0.18	1.60 (30)	0.79	1.15 (45)	0.25	1.23 (40)	0.33			1.10 (30)	0.37	1.23 (10)	0.13	1.06 (5)	ND
	Middle	0.98 (55)	0.36	0.47 (10)	0.28	0.79 (10)	0.37	1.28 (30)	0.14	0.86 (45)	0.14	0.70 (35)	0.22	0.56 (15)	0.20	0.76 (25)	1.98	0.81 (10)	0.22	0.84 (5)	ND
	Aplical	0.75 (55)	0.18	0.21 (10)	0.02	0.41 (10)	0.01	0.91 (30)	0.12	0.64 (45)	0.11	0.49 (10)	0.11			0.55 (35)	0.46	0.51 (10)	0.13	0.48 (5)	ND
Distal	Coronal	1.03 (55)	0.24	1.01 (20)	0.16	1.07 (10)	0.12	1.60 (30)	0.27	1.10 (45)	0.33	1.05 (35)	0.25			1.99 (35)	0.20	1.34 (10)	0.07	0.88 (5)	ND
	Middle	0.63 (55)	0.20	0.47 (10)	0.16	0.51 (10)	0.15	1.18 (30)	0.23	0.74 (45)	0.23	0.43 (35)	0.15	0.62 (15)	0.30	0. 90 (25)	0.10	0.94 (10)	0.16	0.49 (5)	ND
	Aplical	0.59 (55)	0.27	0.29 (10)	0.17	0.58 (10)	0.11	0.75 (30)	0.14	0.55 (45)	0.17	0.21 (10)	0.20			0.68 (35)	0.09	0.63 (10)	0.08	0.23 (5)	ND
Palatal/Lingual	Coronal	1.44 (35)	0.39	1.19 (20)	0.22	2.20 (10)	0.50	1.82 (30)	0.34	1.24 (10)	0.40	1.46 (35)	0.26			1.49 (25)	0.24	1.68 (10)	0.25	1.24 (5)	ND
	Middle	0.74 (40)	0.47	1.28 (15)	0.46	1.45 (10)	0.23	1.28 (30)	0.40	2.11 (10)	1.33	0.86 (35)	0.40	0.82 (25)	ND	1.05 (15)	0.18	1.32 (10)	0.05	0.96 (5)	ND
	Aplical	0.76 (45)	0.20	0.96 (10)	0.32	0.85 (10)	0.04	1.08 (30)	0.43	1.34 (35)	0.55	0.43 (10)	0.08			1.19 (25)	0.98	0.81 (10)	0.11	0.39 (5)	ND

MV root, mesiobuccal root; DV root, distobuccal root; P root, palatal root; CMV, mesiobuccal canal; CML, mesiolingual canal; CDV, distobuccal canal; CP, palatal canal; CMM, mesiomediobuccal canal; CDL, distolingual canal; ND, no data; SD, standard deviation. MBC, mesio-buccal canal; MLC, mesio-lingual canal; DBC, disto-buccal canal; PC, palatal canal; MMC, mesio-medio canal; DLC, disto-lingual canal; LC, lingual canal.

* Statistically significant difference of means at *p* < 0.05.

In the analysis of the average section of the upper molars, in the palatal root, the average vestibular thickness is 0.70 mm, and in the distobuccal root, the mesial thickness is 0.79 mm with a moderate variability in the cases for both. In the distal thickness for the mesiovestibular root, a higher average thickness is found in the mesiovestibular canal (*x* = 0.63 mm) than in the mesiolingual canal (*x* = 0.47 mm).

In the lower molars, in the analysis of the median section, it can be highlighted that for the distal thickness in the mesiovestibular root there are three canals with the following means: (1) mesiovestibular canal (*x* = 0.74 mm); (2) mesiolingual canal (*x* = 0.62 mm) and (3) mesiomediovestibular canal (*x* = 0.43 mm), for the latter a high variability is found in the collected cases. In the distovestibular root, the high variability of thicknesses in the distovestibular canal (*x* = 0.76 mm) stands out, and the mean in the distolingual (*x* = 0.81 mm) is greater than in the previously mentioned.

Finally, it should be noted that there is an anomalous case where a lingual root is found in the lower molars.

### 3.2 Diameter

Table 2 shows the results obtained. The existence of statistically significant differences between the types of canals for the same roots between upper and lower molars was analysed. Differences with a significance level of less than 0.05 were found in the following cases for the vestibular-lingual diameter and in the mesiobuccal root: in the coronal section, there is a significantly greater mean in the mesiolingual canal in the lower molars (*x* = 1.11 mm) than in the upper ones (*x* = 0.35). Similarly, in the apical section, the mean of the mesiobuccal canal is statistically greater in the lower molars (*x* = 0.65 mm) than in the upper ones (*x* = 0.32 mm). While in the mesiodistal diameter, and in the mesiovestibular root, the following differences are given: in the coronal section, the mean of the mesiolingual canal is statistically greater in the lower molars (*x* = 0.35 mm) than in the upper molars (*x* = 0.17 mm); likewise, in the mean section for the mesiovestibular canal, the mean is significantly greater in the lower molars (*x* = 0.53 mm) than in the upper molars (*x* = 0.36 mm).

**Table 2:** Diameter of the canals.

		Maxillary primary molars								Mandibular primary molars									
		MB ROOT				DB ROOT		P ROOT		M ROOT				D ROOT				L ROOT	
		MBC		MLC		DBC		PC		MBC		MLC		DBC		DLC		LC	
	Section	$\bar{X}(n)$	SD	$\bar{X}(n)$	SD	$\bar{X}(n)$	SD	$\bar{X}(n)$	SD	$\bar{X}(n)$	SD	$\bar{X}(n)$	SD	$\bar{X}(n)$	SD	$\bar{X}(n)$	SD	$\bar{X}(n)$	SD
BL Diameter	Coronal	1.54 (55)	0.71	0.35* (20)	0.14	1.60 (10)	0.26	0.70 (30)	0.41	1.55 (45)	0.86	1.11* (30)	0.45	2.30 (35)	1.25	1.21 (10)	0.85	0.49 (5)	ND
	Middle	0.93 (55)	0.47	0.41 (5)	ND	0.92 (10)	0.01	0.58 (30)	0.36	1.29 (45)	0.52	1.15 (40)	0.42	1.98 (35)	1.48	1.29 (10)	1.04	0.44 (5)	ND
	Aplical	0.32* (55)	0.14	0.22 (10)	0.12	0.65 (10)	0.07	0.40 (30)	0.15	0.65* (45)	0.23	1.38 (10)	0.49	1.06 (35)	0.65	1.56 (10)	0.35	0.29 (5)	ND
MD Diameter	Coronal	0.42 (55)	0.36	0.17* (20)	0.05	0.13 (10)	0.31	1.70 (30)	0.61	0.54 (45)	0.22	0.35* (30)	0.14	0.43 (35)	0.11	0.47 (10)	0.09	0.57 (5)	ND
	Middle	0.36* (55)	0.41	0.18 (5)	ND	0.29 (10)	0.21	1.21 (30)	0.55	0.53* (45)	0.29	0.30 (40)	0.24	0.30 (35)	0.09	0.29 (10)	0.02	0.41 (5)	ND
	Aplical	0.31 (55)	0.23	0.15 (10)	0.05	0.21 (10)	0.06	0.56 (30)	0.16	0.33 (45)	0.21	0.12 (10)	0.01	0.23 (35)	0.09	0.19 (10)	0.07	0.24 (5)	ND

ND, no data; SD, standard deviation; BL, bucco-lingual; MD, mesio-distal; MBC, mesio-buccal canal; MLC, mesio-lingual canal; DBC, disto-buccal canal; PC, palatal canal; MMC, mesio-medio canal; DLC, disto-lingual canal; LC, lingual canal; VL, vestibulolingual; MD, mesiodistal.

* Statistically significant difference of means at *p* < 0.05.

### 3.3 Canal length

In the analysis of the length of the canals, it can be highlighted for the lower molars: the mesial root is longer than the distal root for the first temporary molars (Table 3). While, for the second lower molars, the opposite occurs. In the upper molars, for the first molar, data are only found in the mesiovestibular root with an average length of 6.15 mm; while, for the second molars, the upper average is in the mesiovestibular root with a value of 8.13 mm, followed by the palatal root with 6.65 mm.

**Table 3:** Canal length.

Primary molars	*n*	Root	Mean canal lenght (mm)	
			$\bar{X}$	SD
Mandibular				
First Molar	20	M (*n* = 20)	7.23	2.04
		D (*n* = 10)	4.71	1.66
		L (*n* = 5)	10.77	ND
Second Molar	30	M (*n* = 25)	6.62	1.54
		D (*n* = 25)	6.88	3.64
Maxillary				
First Molar	20	MB (*n* = 20)	6.15	1.85
		DB (*n* = 0)	ND	
		P (*n* = 0)	ND	
Second Molar	50	MB (*n* = 35)	8.13	2.91
		DB (*n* = 10)	5.52	0.83
		P (*n* = 30)	6.65	1.73

M, mesial; D, distal; l, lingual; MB, mesio-buccal; DB, disto-buccal; P, palatal; 1MT, first temporary molar; 2MT, second temporary molar; RM, mesial root; RD, distal root; RL, lingual root; RMV, mesiovestibular root; RDV, distovestibular root; RP, palatal root; ND, no data.

### 3.4 Vertucci classification

Table 4 shows the predominant root type for the analysed teeth according to Vertucci's classification. For the upper molars, the predominant root is palatal with a type I classification, followed by the mesiovestibular root with a type I classification. However, in the lower molars, the predominant root is the mesial with a type IV classification, followed by the distal root type I.

**Table 4:** Vertuccìs classification.

Vertucci type	Maxillary primary molars (*n* = 70)			Mandibular primary molars (*n* = 50)		
	MBR (*n* = 55)	DBR (*n* = 10)	PR (*n* = 30)	MR (*n* = 45)	DR (*n* = 35)	LR (*n* = 5)
I	20	5	30	5	15	5
II	5					
III	15	5			5	
IV				20	5	
V	10				5	
VI	5					
VII				10		
VIII						
Not classifiable				10	5	

MBR, mesio-buccal root; DBR, disto-buccal root; PR, palatal root; MR, mesial root; DR, distal root; LR, lingual root.

### 3.5 Isthmus

In the upper molars, in general, it can be observed that they appear in the mesiovestibular root, specifically there is more presence in the coronal and middle section, than in the apical section (Table 5). When analysing the lower molars, generally, isthmus can be found both in the mesial and distal roots. In the mesial root, isthmus were found in the three analysis sections (coronal, middle and apical), while in the distal root there were more cases in the middle section.

**Table 5:** Presence of isthmus by root.

	Maxillary temporary molars (*n* = 70)			Mandibular temporary molars (*n* = 50)		
	MBR (*n* = 55)	DBR (*n* = 10)	PR (*n* = 30)	MR (*n* = 45)	DR (*n* = 35)	LR (*n* = 5)
Presence of isthmus						
Yes	45	5	5	45	20	0
No	10	5	25	0	15	5
Coronal third						
Yes	20	5	5	35	15	0
No	35	5	25	10	20	5
Middle third						
Yes	30	5	0	40	20	0
No	25	5	30	5	15	5
Apical third						
Yes	25	0	0	45	15	0
No	30	10	30	0	20	5

Presence and location of isthmus in the sections of the roots of primary temporary molars. MBR, mesio-buccal root; DBR, disto-buccal root; PR, palatal root; MR, mesial root; DR, distal root; LR, lingual root.

### 3.6 Lateral canals and foramina

Table 6 analyses the number of lateral canals, foramina and the number found in each section for type of root in upper or lower molars. Regarding the lateral canals, the mesiobuccal root stands out in upper molars, where there are generally between 0 and 1 canals, which end in the same number of foramina; specifically, there are more cases of canals for this root in the apical section, given that in the coronal and middle sections the number of canals is 0. Likewise, in the palatal root in the apical Section 1 or 3 canals and foramina appear. Focusing the analysis on the lower molars, in the case of the distal root of the 7 cases studied, only in 1 are 3 lateral canals and foramina found in the three sections. In the mesial root, varied cases of both canals and foramina appear and in general, the appearance of canals is more predominant in the apical and middle sections.

**Table 6:** Lateral canals and foramina.

	Maxillary primary molars (*n* = 70)			Mandibular primary molars (*n* = 50)		
	MBR (*n* = 55)	DBR (*n* = 10)	PR (*n* = 30)	MR (*n* = 45)	DR (*n* = 35)	LR (*n* = 5)
Number of lateral canals						
0	15	5	5	10	25	5
1	20	0	15	10	0	0
2	5	0	0	10	0	0
3	10	5	10	5	5	0
>3	5	0	0	10	5	0
Number of foramina						
0	15	5	5	10	25	5
1	20	0	15	10	0	0
2	5	0	0	10	0	0
3	10	5	10	5	5	0
>3	5	0	0	10	5	0
Coronal third						
0	50	5	25	40	30	5
1	5	0	0	5	0	0
2	0	0	0	0	5	0
3	0	5	5	0	0	0
>3	0	0	0	0	0	0
Middle third						
0	50	5	25	30	25	5
1		5	5	5	5	0
2	5	0	0	5	5	0
3		0	0	5	0	0
>3		0	0		0	0
Apical third						
0	5	2	15	10	25	5
1	3	0	10	20	0	0
2	0	0	0	10	10	0
3	2	0	5	5	0	0
>3	1	0	0	0	0	0

Total number of lateral canals and foramina. Lateral canals *y* foramina location in the sections of the roots of primary molars. MBR, mesio-buccal root; DBR, disto-buccal root; PR, palatal root; MR, mesial root; DR, distal root; LR, lingual root.

## 4 Discussion

The root anatomy of primary teeth has been studied using different techniques. Initially, conventional radiographs were used, and then, to obtain more detailed results, the clearing technique was used (28, 30, 31), electron microscopes (27, 32), until reaching the most current techniques, CBCT (5, 21, 29, 33, 34), MDCT scanner (2) and finally, the Micro-CT (16, 18, 23, 24, 26–28, 35).

The use of Micro-CT, although limited to *in vitro* studies, is essential in the dental field, especially in endodontics. Thanks to the high resolution and precision of the 3D images it produces, its help is decisive for the development of clinical research, anatomical diagnosis and updating of clinical procedures and protocols for pulp treatments (36). Grande et al. reported that the use of Micro-CT must be valued as “the standard analytical reference method to study and determine the morphology of root canals” (37).

Micro-CT images allow for a precise and comprehensive description of the different root canal systems, as well as helping professionals in the selection of materials and instruments for endodontic treatment (36). Mohd Ariffin et al. (18) reported that no way has been found to use Micro-CT at a clinical level in patients; this would provide significant help and information to professionals to correctly perform treatments, and at the same time improve the prognosis of this.

This study analyses the pulp canal system of primary teeth taking into account the Vertucci classification. We agree on the parameters studied with some authors (18, 24, 26, 35) but, not all studies have used this classification, in fact, some consider other criteria (2, 16, 23, 27–29, 32, 34). The results obtained are reported in Table 7.

**Table 7:** Reviewed articles that take into account the anatomy of primary molars.

Autor	Year	Number of teeth analysed	Primary molars	Study technique used	Vertucci Classification	Other criteria
Fumes et al. (16)	2014	40	10 Maxillary First molar	Micro-CT	No	Dentine thickness
			10 Maxillary Second molar			Location of canals
			10 Mandibular First molar			Number of canals
			10 Mandibular Second molar			Canals volume
						Canals area
						SMI
						Canals diameter
						Canals roundness
						Canals length
						Root length
El Hachem et al. (24)	2019	10	Mandibular Second molar	Micro-CT	Yes	Presence and location of lateral canals
						Presence and location of isthmuse
						Canals length
						BL and MD diameter of canals
						Dentine thickness
						Direction of minimum dentine thickness
Mohd Ariffin et al. (18)	2020	57	Maxillary Second molar	MiCro-CT	Yes	DV and P root fusion
Datta et al. (2)	2019	64	16 Maxillary First molar	MDCT	No	Number of roots
			16 Maxillary Second molar	Multidetector computed tomography		Number of canals
			16 Mandibular First molar			Root length
			16 Mandibular Second molar			
Ozacan et al. (19)	2015	343	81 Maxillary First molar	CBCT	Yes	Number and morphology of roots
			100 Maxillary Second molar			Number of canals
			72 Mandibular First molar			Roundness and shape of the canals
			90 Mandibular Second molar			Canals length
						Root length
						Clasification in 8 types
Wang et al. (23)	2013	29	8 Maxillary First molar	Micro-CT	No	Number of roots
			10 Maxillary Second molar			Number of canals
			2 Mandibular First molar			DV and P root fusion
			9 Mandibular Second molar			Canals shape
Yang et al. (34)	2013	487	Mandibular Second molar	CBCT	No	Number and morphology of roots
						Number and morphology of canals
Bandeira et al. (27)	2021	16	4 Maxillary First molar	Electronic microscope and Micro-CT	No	Frequency of accessory canals
			4 Mandibular First molar			Accessory canals shape
			4 Maxillary Second molar			Accessory canals diameter
			4 Mandibular Second molar			Accessory canals type
Kumar (32)	2016	60	Maxillary First molar	Electronic microscope	No	Number of accessory canals
			Maxillary Second molar			Accessory canals shape
			Mandibular First molar			Accessory canals diameter
			Mandibular Second molar			
Bagherian et al. (31)	2010	90	27 Mandibular First molar	Clearing technique	Yes	Number of roots
			27 Maxillary First molar			Root Shape and Roundness
			22 Mandibular Second molar			Root length
			14 Maxillary Second molar			Root angulation
						Number of canals
Rahmati et al. (33)	2023	60	Maxillary First molar	CBCT	Yes	Number of canals
			Maxillary Second molar			Frequency and distribution of root concavity
			Mandibular First molar			Number of roots
			Mandibular Second molar			
Ticona-Flores et al. (29)	2022	132	30 Maxillary First molar	CBCT	No	Weine Clasification
			23 Maxillary Second molar			Root length
			44 Mandibular First molar			Canals length
			35 Mandibular Second molar			Canals angulation
						Canals volume and surface
						Canals diameter
Acar (28)	2015	41	20 Maxillary molars	Micro-CT, clearing technique and CBCT	No	Accessory canals
			21 Mandibular molars			
Katge et al. (30)	2018	120	30 Maxillary First molar	Clearing technique	Yes	Number of canals
			30 2MT SUP			Canals curvature
			30 Mandibular First molar			
			30 Mandibular Second molar			
Teixeira et al. (26)	2023	60	30 Maxillary molars	Micro-CT	Yes	Number of roots
			30 Mandibular			Number of canals
						Rooth curvature
						Presence of lateral canals
						Dentine thickness at furcation
						SMI
						Canals Volume
						Canals surface
Demiriz et al. (21)	2017	228	Mandibular Second molar	CBCT	Yes	Root shape
Meryem et al. (35)	2019	50	17 Mandibular First molars	Micro-CT	Yes	None
			33 Mandibular Second molars			

According to our results, we agree with Mohd Ariffin et al. (18), Ozcan et al. (19) and Teixeira et al. (26) on the morphology of the palatal root canal of upper molars: in all the roots studied, only one canal is present, which is considered Type I of the Vertucci classification. Mohd Ariffin et al. (18), analyses only the upper second molars, and also makes another subdivision between second molars with separated roots and fused roots, that is, the distovestibular and palatal roots joined: in this case it is classified as a single canal system, and the most frequent is Type V. In molars with three separated roots, Type I is the most frequent in the palatal and distovestibular roots, while for the mesiovestibular root Type V followed by Type I. Likewise, Ozcan et al. (19) analyses temporary upper second molars with fusion of the palatal root with the distovestibular root, and in their study, also, Type V is the most frequent in this type of root. Root fusion is an anatomical variation that can occur between two or more roots, and in the literature, there is no specific prevalence of fusion between the palatal and distovestibular roots in temporary teeth; Although it has been seen that it is more frequent in upper first molars than in second molars (23).

Likewise, Mohd Ariffin et al. state that the physiological root resorption that occurs in deciduous teeth transforms and changes the morphology of the root canal system. Therefore, it is possible to incur errors in the classification of the canals: for example: a Vertucci Type V can be considered Type I because root resorption has begun (18).

In the other side, Teixeira et al. analysed upper and lower molars, differentiating between first and second. In the mesial root of the mandibular first molars, types IV and V are the most frequent, and in the distal root type I. In the mandibular second molars, the mesial root predominantly presents type V and the distal root types I and II (26). Whereas, according to Merymen et al. (35), type IV is the most frequent in the lower first molars: 47% in the mesial root and 41.2% in the distal root.

In our study, as regards the mesiobuccal root of upper molars, Type I is the most frequent morphology, followed by Type III and Type V. Type II and Type VI are present in one specimen each. In contrast, in the distobuccal root, it can be seen that Type I and Type III are the only ones found, also due to the scarcity of samples of this type of root. In mandibular molars, the distal root presents a varied morphology of the root canals, while, in the mesial root, Type IV is the most frequent, followed by Type VII and finally Type I. In their study, El Hachem et al. (24) examined only lower second primary molars: the type of canal system that is mostly located in the mesial roots is Type IV, while in the distal ones it is Type V.

**Figure 6:**
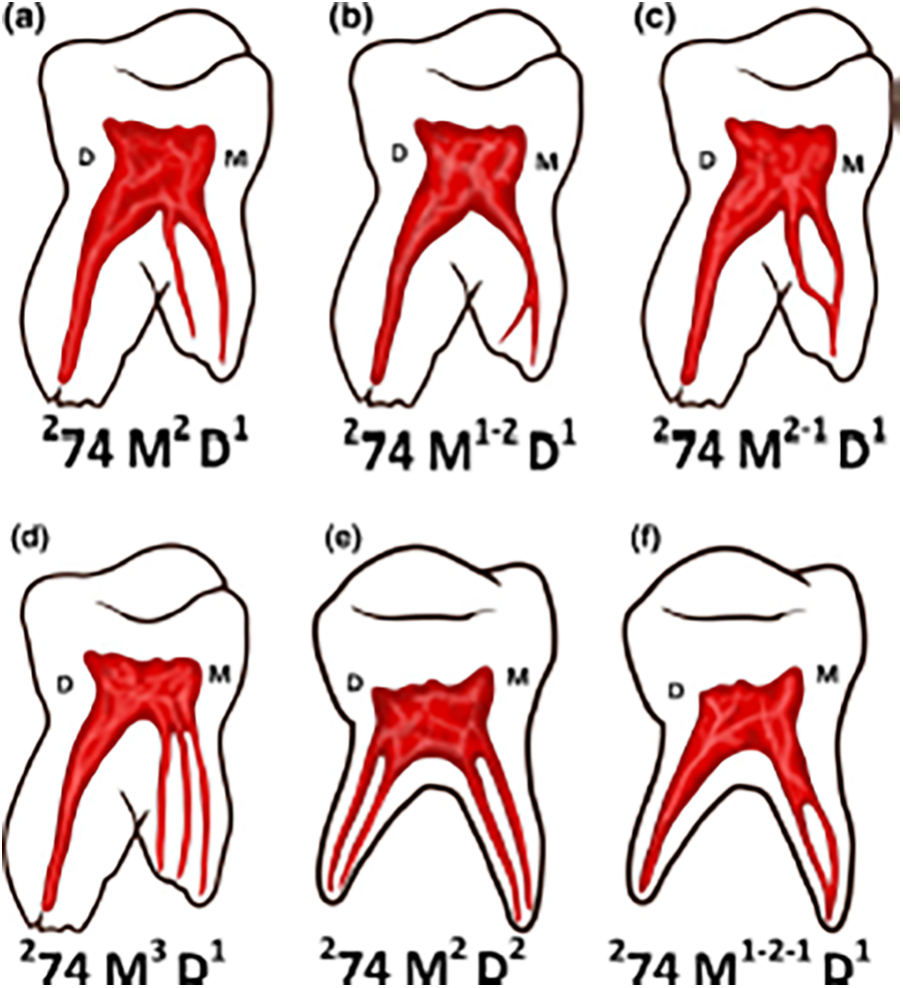
Lower primary molars with their canal system identified according to Ahmed's classification. This classification uses the tooth number in any numbering system, the number of root and the canal system present in each root. **(a)** 274 M2 D1: 2: two roots; 74: primary left mandibular first molar; M2: mesial root with 2 canals; D1: distal root with one canal. **(b)** 274 M1-2 D1: 2: two roots; 74: primary left mandibular first molar; M1: mesial root starts with 1 canal and ends with two canals; D1: distal root with one canal. **(c)** 274 M2-1 D1: 2: two roots; 74: primary left mandibular first molar; M2-1: mesial root starts with two canals and ends with 1 canal; D1: distal root with one canal. **(d)** 274 M3 D1: 2: two roots; 74: primary left mandibular first molar; M3: mesial root with 3 canals; D1: distal root with one canal. **(e)** 274 M2 D2: 2: two roots; 74: primary left mandibular first molar; M2: mesial root with two canals; D2: distal root with two canals. **(f)** 274 M1-2-1 D1: 2: two roots; 74: primary left mandibular first molar; M1-2-1: mesial root starts with one canal, then two canals and ends with one canal; D1: distal root with one canal.

Ahmed et al. (22) presented a new method of classification for primary dentition. This classification uses the tooth number in any numbering system, the number of roots and the canal system present in each root. The root number is placed before the tooth number as a superscript, while the canal system is always added as a superscript, but to the right after the tooth number. If the tooth has more than one root, a letter identifying each root present must be added; for example: lower right first molar is 284 M2 D1, where “M” stands for mesial root and “D” stands for distal root (Figure 6). The superscript numbers to the right of the letters mean the number of canals in the root. Furthermore, they add further information on the root canal system by providing data on the location of accessory canals (22). The root is divided into three parts: coronal (C), middle (M) and apical (A), each letter is written as a superscript in parentheses after the tooth number and after the root number22. If the accessory canal is located in the floor of the pulp chamber, the number is put as a superscript, but to the left of the root letter (22). To indicate apical delta, the letter “D” is used. Abbreviations in parentheses are used before the tooth number to indicate the presence of anomalies in the tooth: for example, (DE) is used for dens evaginatus and (RF) to indicate root fusion (22).

**Figure 7:**
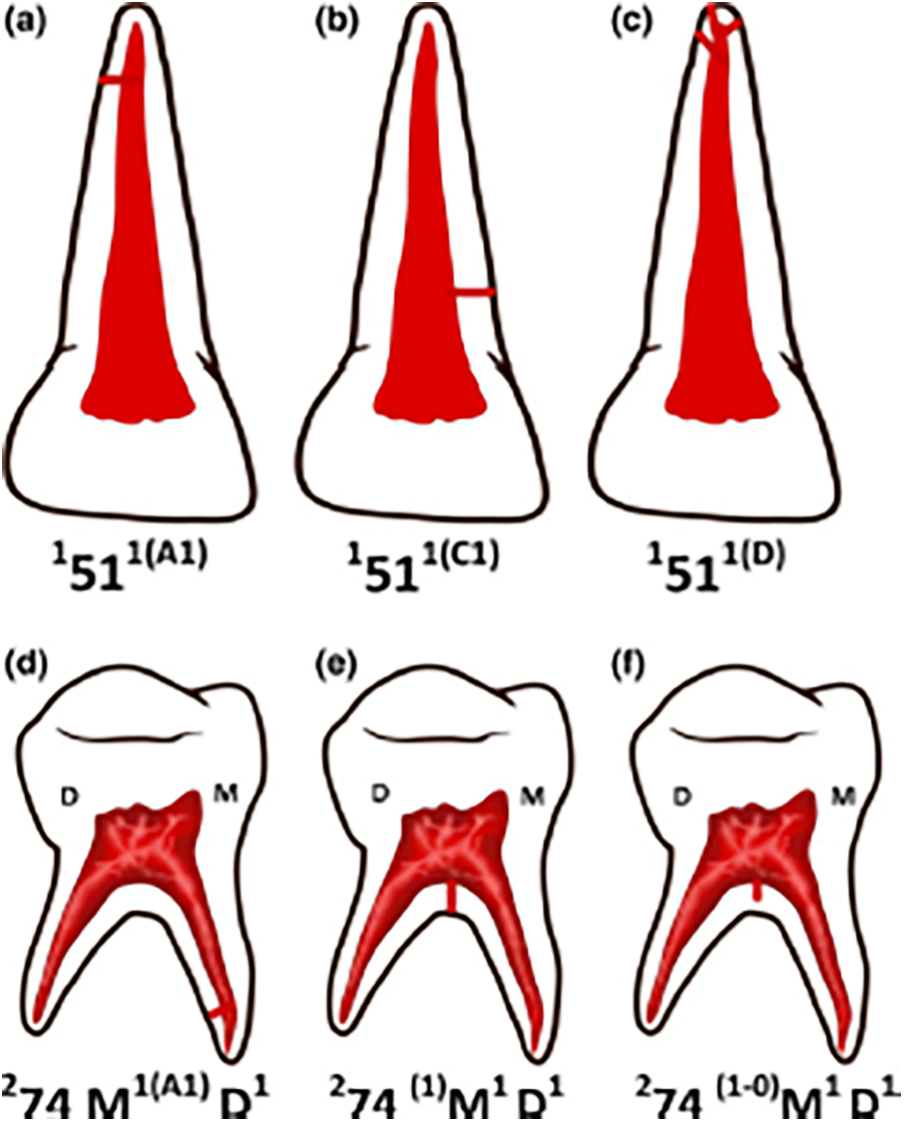
Picture of primary teeth according to Ahmed's classification with information on lateral canals. The root is divided into three parts: coronal (C), middle (M) and apical (A), each letter is written as a superscript in parentheses after the tooth number and after the root number22. If the accessory canal is in the floor of the pulp chamber, the number is put as a superscript, but to the left of the root letter. **(a)** 151 1(A1): 1: one root; 51: primary right maxillary central incisor; 1: one canal; (A1): at apical level one lateral canal. **(b)** 151 1(C1): 1: one root; 51: primary right maxillary central incisor; 1: one canal; (C1): at coronal level one lateral canal. **(c)** 151 1(D): 1: one root; 51: primary right maxillary central incisor; 1: one canal; (D): apical delta. **(d)** 274 M1(A1) D1: 2: two roots; 74: primary left mandibular first molar; M1: mesial root with one canal; (A1): at apical level one lateral canal; D1: distal root with one canal. **(e)** 274 (1) M1 D1: 2: two roots; 74: primary left mandibular first molar; (1): one accessory canal in the pulp chamber that ends in the furcation; M1: mesial root with one canal; D1: distal root with one canal. **(f)** 274 (1 -0) M1D1: 2: two roots; 74: primary left mandibular first molar; (1 -0): one accessory canal in the pulp chamber, it's a blind canal. M1: mesial root with one canal; (A1): at apical level one lateral canal; D1: distal root with one canal.

According to Vertucci's classification, in the present study three roots could not be classified, which had two canals in the coronal, three in the middle third, and two in the apical third. Whereas, with Ahmed's classification it could have been identified in this way: 285 M232 D232. As can be seen in Figure 7, after the letter that indicates the root, the number of canals from coronal to apical that the root has can be put.

In this study, a lower first molar was found with three roots: mesial root, distal root and distolingual root. The root has a long canal length, and is Type I according to Vertucci. Several authors (16, 31) claimed that anatomical changes, such as the number of roots and canals, can be related to genetic, gender and ethnic aspects: these criteria were not taken into account in the study presented here.

Fumes et al. (16) using Micro-CT, considered the thickness of the dentin in the apical third of the maxillary and mandibular primary molars: at 1, 2 and 3 mm from where root resorption begins. There was no statistical difference when comparing the external and internal dentin of the two groups of molars: in both, on the internal surface of the roots, the thickness of the dentin was less. On the other hand, the greatest thickness of the dentin was found in the distal and palatal roots of the upper and lower molars. In this study, the thickness of the dentin is minimum on the furcation side in all axial sections. In addition, the thickness of the dentin decreases from coronal to apical due to the physiological resorption of the primary molars. While in the study by Teixeira et al. (26) the thickness of the dentin between the floor of the pulp chamber and the outer portion of the furcal was analysed using a linear tool: the results were 1.53 mm in the upper molars and 1.59 mm in the lower molars. It should be noted that, in their analysis of dentin thickness, Teixeira et al. (26) did not explain in detail how the measurement was made, nor did they draw up a table with the values found. In our study, three heights were set in an axial direction, and the thickness of the dentin was measured from the inner walls of the canal to the outermost point of the dentin in the vestibular, mesial, lingual and distal directions. Since there is no single protocol for assessing dentin thickness, the studies cannot be entirely comparable.

The fact that temporary teeth undergo physiological resorption affects the thickness of the dentin. In the analysis by Fumes et al. (16), and also in our study, there was a decrease in the thickness of the dentin in the apical section, compared to the middle and coronal sections, especially on the internal surfaces of the roots. When performing pulpectomy on primary molars, using endodontic files to shape the root canal, the following must be taken into account: the shape of the canal, the lesser thickness of the dentin walls, and also, the possible change in location of the apical foramen.

For El Hachem et al. (24), the minimum dentin thickness is found in the middle section at the level of the distal canal and the mesiobuccal canal. On the contrary, in the mesial root, the minimum apical dentin thickness is greater in the mesiobuccal and mesiolingual canals. In the distal root with two canals, the minimum apical thickness is less. While, at the coronal level, there were no differences between all the canals (24). According to the author, due to the presence of the germ of the permanent tooth that is housed between the two roots of the temporary molars, it can be seen that, in the middle section, the thickness of the root dentin in the distal wall of the mesial root and in the mesial wall of the distal roots is much less with respect to the apical and coronal sections (24).

It was decided to measure the thickness of the dentin because one of the major problems in endodontic treatment is lateral perforation during instrumentation of the canals (24). The walls that must be treated with the greatest care when performing the pulpectomy treatment with files are those that are most exposed to physiological resorption: the vestibular surface of the palatal root, the distal surface of the mesial root and the mesial surface for the distal root. In fact, excessive thinning of these dentin walls of the root canals can cause fractures. As Tomer et al. (15) also say, during the chemical-mechanical preparation of the canals, the minimum thickness required, which must remain along the length of the canal, is 1 mm. After mechanical or manual instrumentation, it is essential that the thickness of the remaining dentin is sufficient to withstand occlusal and lateral forces since if the removal of dentin from the walls is very aggressive, exfoliation of the deciduous tooth can be accelerated (38).

The thickness of the dentin is related to the diameter of the root canals, since it was measured in the same axial section. El Hachem et al. (24) stated that the vestibulolingual diameter is greater in all three sections, in the distal root that only has one pulp canal. In the present study, in the distal root of the lower molars, two canals were observed, the distobuccal canal and the distolingual canal; the mean vestibulolingual diameter of the distobuccal canal is the largest of all the roots, in the three sections. While for the mesiodistal diameter, the palatal root is the one with the highest mean.

According to some authors (39), the lateral canal decay is considered to be the cause of interradicular pathology of deciduous molars, considering that it is almost impossible to clean them during endodontic treatment. For this reason, their presence and location are also taken into account in our study. Bandeira et al. (27), founded that their frequency is higher in upper molars. El Hachem et al. (24) managed to identify the number of lateral canals present in the lower temporary molars that they studied: in all mesial roots there are two lateral canals, while in the distal root there are two lateral canals or one, and they are mainly found in the middle and apical third. Also, Sharma et al. (40) stated that, in temporary teeth, most of the accessory canals are found in the furcation area; although in their opinion, the presence of these canals is not the main cause of infections or pulpectomy failure.

In our study, most of the lateral canals are located in the apical section of the mesiobuccal root of the upper molars and in the distal root of the lower molars, as also stated by El Hachem et al. (24). Similar results were also found by Teixeira et al. (26) who found the lateral canals mainly in the middle section and in the apical section of the lower molars and only in the apical area for the upper molars, although they do not specify in which root they were found. Bandeira et al. (27) reported that there is a higher frequency of accessory canals in upper molars, although their sample is smaller than in other studies. All the lateral canals found in our analysis by Micro-CT end with foramina, only Bandeira et al. (27) analysed the terminations of the accessory canals: in their publication they differentiated them into blind canals, with inter-canal communications or with contact with the periodontium. While the other articles considered (24, 26) speak only of location.

According to Zoremchhingi et al. (41), all mesial roots present isthmus in the coronal section, but none in the apical section. In the distal roots, however, the isthmus is maintained in 28.6% of the roots. The diameter of the canals decreases progressively from coronal to apical. The presence of isthmus is very important when performing endodontic treatment (42–44). In the present study, it was possible to see that isthmus were present in mesiobuccal roots of upper molars and in mesial roots of lower molars. These results are consistent with those of El Hachem et al. (24), where all mesial roots of lower molars present isthmus in the coronal third, but not in the apical third, although in our study there is presence of isthmus in the apical section. This may be due to the fact that in the studies two different apical cutting heights are evaluated.

As regards the length of the root canals, in our study it was measured from two pre-established points: from 1 mm below the furcation to 1.5 mm from the anatomical apex. The average of the longest canal is that of the mesiobuccal root of the second upper molar with 8.13 ± 2.91 mm. Whereas, in the lower molars, it turns out that the average of the mesial roots of the first temporary molars is the longest, although the longest root with 10.77 mm is the lingual root, which was only found in one specimen, and is considered an anatomical variation. Other authors such as Fumes et al. (16) calculated the length from the cementoenamel junction to the apical foramen, so it is not comparable. It is necessary to protocolise the measurement in order to perform a correct and reliable comparison examination, which results in a limitation for this study and subsequent ones.

## 5 Conclusion

The root pulp anatomy of temporary teeth is very complex and varied and more studies are needed on it, requiring a more precise classification that covers all the anatomical variation that the teeth may present.

The use of Micro-CT is essential for the detailed analysis of the internal structures of temporary and permanent teeth. The high costs and the inability to use *in vivo* studies are some of the limitations.

The root anatomy of primary teeth is complex, but it is necessary to understand it in order to carry out the necessary treatments depending on the pathology that is present. This study showed the complexity and variability of the root canal morphology of primary molars.

## Data Availability

The original contributions presented in the study are included in the article/Supplementary Material, further inquiries can be directed to the corresponding author.

## References

[1] Cleghorn BM Boorberg NB Christie WH. Primary human teeth and their root canal systems. Endod Topics. (2010) 23:6–33. 10.1111/etp.12000

[2] Datta P Zahir S Kundu GK Dutta K. An in vitro study of root canal system of human primary molars by using multidetector computed tomography. J Indian Soc Pedod Prev Dent. (2019) 37:120–6. 10.4103/1319-2442.261339 31249173

[3] Koshy S Love RM. Endodontic treatment in the primary dentition. Aust Endod J. (2004) 3:59–68. 10.1111/j.1747-4477.2004.tb00183.x 15378974

[4] World Health Organization. Global Oral Health status Report: Towards Universal Health Coverage for Oral Health by 2030. Geneva: World Health Organization (2022).

[5] Ounsi HF Debaybo D Salameh Z Chebaro A Bassam H. Endodontic considerations in pediatric dentistry: a clinical perspective. Int Dent South Afr. (2009) 11:40–50.

[6] Abanto J Tsakos G Olegário IC Paiva SM Mendes FM Ardenghi TM Impact of pulpectomy versus tooth extraction in children’s oral health-related quality of life: a randomized clinical trial. Community Dent Oral Epidemiol. (2024) 52:13–23. 10.1111/cdoe.12895 37519111

[7] Chen X Liu X Zhong J. Clinical and radiographic evaluation of pulpectomy in primary teeth: a 18-months clinical randomized controlled trial. Head Face Med. (2017) 13:12. 10.1186/s13005-017-0145-1 29073902 PMC5658955

[8] Aminabadi NA Farahani RM Gajan EB. Study of root canal accessibility in human primary molars. J Oral Sci. (2008) 50:69–74. 10.2334/josnusd.50.69 18403887

[9] Katge F Dixit UB. Root and root canal anatomy of primary mandibular central incisor, lateral incisor, and canine in Indian children: a cone beam computed tomography study. Int J Dent. (2022) 7:7191134. 10.1155/2022/7191134 35356037 PMC8958108

[10] Krishnamurthy NH Jose S Thimmegowda U Bhat PK. Evaluation of anatomical variations in root and canal morphology of primary maxillary second molars: a cone-beam computed tomography study. Int J Clin Pediatr Dent. (2021) 14:628–32. 10.5005/jp-journals-10005-2030 34934273 PMC8645619

[11] Cortés O Boj JR. Pulp treatments in temporary dentition. In: Boj JR Catalá M Garcia–Ballesta C Mendoza A Planells P, editors. Pediatric Dentistry. The Evolution of The Child to The Young Adult. Madrid: Ed. Ripano (2011). p. 334–50.

[12] Orellana Centeno JE Guerrero Sotelo RN Vasquez Morales R Ruíz Martínez CL. Anatomy of the temporary dentition. Rev Latinoam Orto y Odontop. (2022) 22:1–8.

[13] Haralur SB Al-Qahtani AS Al-Qarni MM Al-Homrany RM Aboalkhair AE. Influence of remaining dentin wall thickness on the fracture strength of endodontically treated tooth. J Conserv Dent. (2016) 19:63–7. 10.4103/0972-0707.173201 26957796 PMC4760017

[14] Nisar P Katge F Bhanushali P Deshpande S Poojari M Shetty S. Comparative in vitro evaluation of remaining dentine thickness following instrumentation with hand and rotary endodontic files during pulpectomy in primary molars: a systematic review. Eur Arch Paediatr Dent. (2023) 24:15–32. 10.1007/s40368-022-00760-4 36319891

[15] Tomer AK Miglani A Chauhan P. An in vitro evaluation of remaining dentine thickness through CBCT using different files. IOSR J Dent Med Sci. (2017) 16:121–4. 10.9790/0853-160201121124

[16] Fumes AC Sousa-Neto MD Leoni GB Versiani MA da Silva LAB da Silva RAB Root canal morphology of primary molars: a micro-computed tomography study. Eur Arch Paediatr Dent. (2014) 15:317–26. 10.1007/s40368-014-0117-0 24563173

[17] Alafandy AS Makieh RE. The difference distance between the apical foramen and the anatomical apex in primary teeth-an in vitro study. Clin Exp Dent Res. (2023) 9:913–21. 10.1002/cre2.784 37703170 PMC10582222

[18] Mohd Ariffin S Dalzell O Hardiman R Manton DJ Parashos P Rajan S. Root canal morphology of primary maxillary second molars: a micro-computed tomography analysis. Eur Arch Paediatr Dent. (2020) 21:519–25. 10.1007/s40368-020-00515-z 32100200

[19] Ozcan G Sekerci AE Cantekin K Aydinbelge M Dogan S. Evaluation of root canal morphology of human primary molars by using CBCT and comprehensive review of the literature. Acta Odontol Scand. (2016) 74:250–8. 10.3109/00016357.2015.1104721 26523502

[20] Ahmed HMA Versiani MA De-Deus G Dummer PMH. A new system for classifying root and root canal morphology. Int Endod J. (2017) 50:761–70. 10.1111/iej.12685 27578418

[21] Demiriz L Bodrumlu EH Icen M. Evaluation of root canal morphology of human primary mandibular second molars by using cone beam computed tomography. Niger J Clin Pract. (2018) 21:462–7. 10.4103/njcp.njcp_85_17 29607858

[22] Ahmed HMA Musale PK El Shahawy OI Dummer PMH. Application of a new system for classifying tooth, root and canal morphology in the primary dentition. Int Endod J. (2020) 53:27–35. 10.1111/iej.13199 31390075

[23] Wang Y-L Chang H-H Kuo C-I Chen S-K Guo M-K Huang G-F A study on the root canal morphology of primary molars by high-resolution computed tomography. J Dent Sci. (2013) 8:321–7. 10.1016/j.jds.2013.04.002

[24] El Hachem C Kaloustian MK Nehme W Ghosn N Abou Chedid JC. Three-dimensional modeling and measurements of root canal anatomy in second primary mandibular molars: a case series micro CT study. Eur Arch Paediatr Dent. (2019) 20:457–65. 10.1007/s40368-019-00426-8 30830644

[25] Verma P Love RM. A micro CT study of the mesiobuccal root canal morphology of the maxillary first molar tooth. Int Endod J. (2011) 44:210–7. 10.1111/j.1365-2591.2010.01800.x 20880136

[26] Teixeira TF da Silva AMP Coutinho TMC Marceliano EFV Dos Santos Miranda ARL Ferreira DC Deciduous molars complexity anatomy revealed by computed microtomography. Eur J Dent. (2024) 18:789–95. 10.1055/s-0043-1772566 37729937 PMC11290920

[27] Bandeira AVL Lima MDM Lima CCB Moura MS Cury AADB Moura LFAD. Topography of primary molar pulp chamber floor: a scanning electron microscopy and micro-computed tomography analysis. Pesqui Bras Pediatric Dentistry Clín Integr. (2021) 21:e0033. 10.1590/pboci.2021.150

[28] Acar B Kamburoğlu K Tatar İ Arıkan V Çelik HH Yüksel S Comparison of micro-computerized tomography and cone-beam computed tomography in the detection of accessory canals in primary molars. Imaging Sci Dent. (2015) 45:205–11. 10.5624/isd.2015.45.4.205 26730367 PMC4697004

[29] Ticona-Flores J Diéguez-Pérez M. Cone-beam computed tomography (CBTC) applied to the study of root morphological characteristics of deciduous teeth: an in vitro study. Int J Environ Res Public Health. (2022) 19:9162. 10.3390/ijerph19159162 35954525 PMC9368647

[30] Katge F Wakpanjar MM. Root canal morphology of primary molars by clearing technique: an in vitro study. J Indian Soc Pedod Prev Dent. (2018) 36:151–7. 10.4103/JISPPD.JISPPD_237_16 29970632

[31] Bagherian A Kalhori KA Sadeghi M Mirhosseini F Parisay I. An in vitro study of root and canal morphology of human deciduous molars in an Iranian population. J Oral Sci. (2010) 52:397–403. 10.2334/josnusd.52.397 20881332

[32] Kumar VD. A scanning electron microscope study of prevalence of accessory canals on the pulpal floor of deciduous molars. J Indian Soc Pedod Prev Dent. (2009) 27:85–9. 10.4103/0970-4388.55332 19736500

[33] Rahmati A Khoshbin E Shokri A Yalfani H. Cone-beam computed tomography assessment of the root canal morphology of primary molars. BMC Oral Health. (2023) 23:692. 10.1186/s12903-023-03414-z 37749546 PMC10521415

[34] Yang R Yang C Liu Y Hu Y Zou J. Evaluate root and canal morphology of primary mandibular second molars in Chinese individuals by using cone-beam computed tomography. J Formos Med Assoc. (2013) 112:390–5. 10.1016/j.jfma.2012.10.008 23927978

[35] Meryem ZI Yüksel BN Şaziye SA. Root canal morphology of mandibular primary molars: a micro-CT study. Cumhuriyet Dent J. (2019) 22:382–9. 10.7126/cumudj.615843

[36] Mazzi-Chaves JF Silva-Sousa YTC Leoni GB Silva-Sousa AC Estrela L Estrela C Micro-computed tomographic assessment of the variability and morphological features of root canal system and their ramifications. J Appl Oral Sci. (2020) 28:e20190393. 10.1590/1678-7757-2019-0393 32049137 PMC6999120

[37] Grande NM Plotino G Gambarini G Testarelli L D'Ambrosio F Pecci R Present and future in the use of micro-CT scanner 3D analysis for the study of dental and root canal morphology. Ann Ist Super Sanita. (2012) 48:26–34. 10.4415/ANN_12_01_05 22456012

[38] Seema T Ahammed H Parul S Cheranjeevi J. Comparative evaluation of dentin removal and taper of root canal preparation of hand K file, ProTaper rotary file, and Kedo S rotary file in primary molars using cone-beam computed tomography. Int J Clin Pediatr Dent. (2020) 13:332–6. 10.5005/jp-journals-10005-1787 33149404 PMC7586477

[39] Wrbas KT Kielbassa AM Hellwig E. Microscopic studies of accessory canals in primary molar furcations. ASDC J Dent Child. (1997) 64:118–22. 9189001

[40] Sharma U Gulati A Gill N. An investigation of accessory canals in primary molars - an analytical study. Int J Paediatr Dent. (2016) 26:149–56. 10.1111/ipd.12178 26146865

[41] Zoremchhingi JT Varma B Mungara J. A study of root canal morphology of human primary molars using computed tomography: an in vitro study. J Indian Soc Pedod Prev Dent. (2005) 23:7–12. 10.4103/0970-4388.16019 15858299

[42] Weller RN Niemczyk SP Kim S. Incidence and position of the canal isthmus. Part 1. Mesiobuccal root of the maxillary first molar. J Endod. (1995) 21:380–3. 10.1016/S0099-2399(06)80605-9 7499980

[43] American Association of Endodontists (AAE). Glossary of Endodontic Terms, 10th ed. Chicago, IL, USA: American Association of Endodontists (2020).

[44] Rodriguez RO Gastelum Zazueta AG Hernandez Molinar Y Cardenas Mariel J Gutierrez Cantu FJ Silva-Herzog Flores D. Incidence and type of isthmuses in human first permanent molars, in vitro evaluation. Int J Morphol. (2017) 35:1280–4. 10.4067/S0717-95022017000401280

